# Mushroom hunting and consumption in twenty-first century post-industrial Sweden

**DOI:** 10.1186/s13002-019-0318-z

**Published:** 2019-08-19

**Authors:** Ingvar Svanberg, Hanna Lindh

**Affiliations:** Institute for Russian and Eurasian Studies, Uppsala University, Box 514, 754 22 Uppsala, Sweden

**Keywords:** Gathering activities, Mushrooming, Modern cuisine, Leisure activities, Urban ethnobiology, Ethnomycology, Wild food

## Abstract

**Background:**

The pre-industrial diet of the Swedish peasantry did not include mushrooms. In the 1830s, some academic mycologists started information campaigns to teach people about edible mushrooms. This propaganda met with sturdy resistance from rural people. Even at the beginning of the last century, mushrooms were still only being occasionally eaten, and mostly by the gentry. During the twentieth century, the Swedish urban middle class accepted mushrooms as food and were closely followed by the working-class people. A few individuals became connoisseurs, but most people limited themselves to one or two taxa. The chanterelle, *Cantharellus cibarius* Fr., was (and still is) the most popular species. It was easy to recognize, and if it was a good mushroom season and the mushroomer was industrious, considerable amounts could be harvested and preserved or, from the late 1950s, put in the freezer. The aim of this study is to review the historical background of the changes in attitude towards edible mushrooms and to record today’s thriving interest in mushrooming in Sweden.

**Methods:**

A questionnaire was sent in October and November 2017 to record contemporary interest in and consumption of mushrooms in Sweden. In total, 100 questionnaires were returned. The qualitative analysis includes data extracted from participant and non-participant observations, including observations on activities related to mushroom foraging posted on social media platforms, revealed through open-ended interviews and in written sources. With the help of historical sources, including earlier studies and ethnographical data collections, a diachronic analysis is given to describe the changes over time.

**Results and discussion:**

During the last 100 to 140 years, Sweden has changed from a mycophobic to a mycophilic society with a passionate interest in the utilization of wild mushrooms. In the late twentieth century, various social institutions connected with mushroom hunting evolved. Evening classes, study circles, clubs, exhibitions, consultants, and a wide array of handbooks promoted this interest. In the early twenty-first century, mushrooming has become widely accepted, especially among the middle class, but also among Swedes in general. The so-called hipster-generation, born in the 1990s, harvests mushrooms due to their interest in producing their own food. This group often uses social media to identify edible species. Most people who go mushrooming gather only a few species. There are, however, some dedicated individuals who have become hobby specialists and who know a wide diversity of taxa. A few study participants reported that they were afraid of not being able to distinguish between poisonous fungi species and edible ones and therefore refrain from picking any wild mushrooms at all. However, they still consume cultivated mushrooms, such as *Agaricus bisporus* (J.E. Lange) Imbach, bought in grocery stores or served in cafes and restaurants.

**Conclusion:**

Swedish society has changed rapidly during the last decades and so has the interest in mushrooming among its members. Throughout the second part of the twentieth century, the flow of information about mushrooms has continued through lecturers, courses, media, exhibitions, and even associations. Walking in forestland is also an important leisure activity for many urban Swedes, and in the early twenty-first century, mushrooming has also become a thriving pastime among people with an urban lifestyle.

## Background

### Historical background

From a historical perspective, mushroom hunting in Sweden is a relatively modern activity [1, 2]. As in other Scandinavian countries, mushrooms were not utilized as traditional food in pre-industrial times [3–6]. In fact, the rural population in the Nordic countries had deep-rooted distrust towards mushrooms as nutrient [7]. The peasantry rejected the mushroom as food, even in times of food shortages [8, 9]. Ethnologist Nils Keyland gives an apt description of the lack of interest in mushrooms as food in rural pre-industrial Sweden: ‘It seems that not even a severe famine could overcome the peasantry’s reluctance and indifference to mushrooms as a nutrient. In the old days they would eat grass and leaves, sawdust and dirt, almost anything but not mushrooms, preferring rather to starve to death’ [10]. Consequently, pre-industrial Scandinavia definitely belongs, with a few exceptions, to what scholars have categorized as the mycophobic or mushroom-despising part of the world [11–14].

As a result, very few species of fungi were recognized with folk names before the industrialization and modernization of Sweden [2, 15]. During the eighteenth and nineteenth centuries, the peasantry lacked an interest in mushrooms as food and did not pay them much attention. Although the macrofungi must have been visible in the landscape, people did not even dare to name separate kinds [16]. Ethnographical sources indicate, however, that they could identify a few fungi taxa which, for various reasons, were part of their folk knowledge of the biota. For instance, fly agaric (*Amanita muscaria* (L.) Lam.) was easily recognized and widely used to kill lice, bedbugs, and house flies [17–19], possibly due to its content of ibotenic acid and muscimol [20]. Hence, its Swedish name is *flugsvamp* (‘fly mushroom’). Called *fluoswamp* in the fifteenth century*,* its use was already known [15, 21].^1^ Another macrofungus, *Phallus impudicus* L., was known by the folk name *trollägg* (‘troll’s egg’) due to its shape [22], but it did not have any known use in Sweden.

In northern Sweden, the peasant hunters used a dry mushroom as bait in squirrel traps [23]. That fungus was therefore called *ekorrsvamp* or *ikorrsopp* (both meaning ‘squirrel fungus’) [22, 24]. Although the species cannot be determined with certainty, mycologist Elias Fries identified it as *Octaviana variegata* Vittad. in the mid-nineteenth century [22]. However, Fries’s identification is made even more uncertain by the fact that *Melanogaster variegatus* (Vittad.) Tul. & C. Tul. (the contemporary accepted name for *O. variegata*) is a rare species in Sweden. Most sources refer only to ‘a piece of dried fungus’ [23, 25]. This practice of using a dry fungus as bait in traps was already recorded in the eighteenth century and was still in use among hunters in northern Sweden in the early twentieth century [26, 27].

Traditionally, some taxa were widely used in folk therapy, especially puffballs (*Bovista*, *Lycoperdon*), not only among Swedes but across the Nordic countries. The puffball’s dust-like spores were sprinkled over wounds, but the ‘dust’ was also feared since it was said to cause blindness according to folk beliefs [3, 16, 17, 28, 29]. Puffballs were known by many often humorous folk names: *blindsvamp* (‘blind fungus’), *kärringsvamp* (‘old woman fungus’), *kärringfis* (‘old woman’s fart’), *Skams snusdosa* (‘the devil’s snuff box’), and *fessvamp* (‘fart-fungus’) [16, 19].

A few other fungi taxa—usually having strange shapes—played an important role in local folklore, such as *Crucibulum leave* (Huds.) Kamble, *Fuligo septic* (L.) F.H. Wigg., *Lycogala epidendrum* (J.C. Buxb. ex L.) Fr., *Mucilago spongiosa* (Leyss.) Morgan, and the dreaded *Claviceps purpurea* (Fr.) Tul., which caused ergotism among the peasantry [19]. The deer truffle, *Elaphomyces granulatus* L., was widely known and used as a kind of aphrodisiac for domestic animals and sometimes women [19, 30].

Several taxa of polypores were also culturally salient and have been utilized in Sweden for health-related, technical, and other purposes [14]. A few rust fungi (*Coleosporiaceae*) have also been part of the local knowledge. One such fungus, *Chrysomyxa woroninii* Tranzschel, which occurs in forests on spruce shots, was appreciated as a kind of snack and refreshment in northern Sweden [1, 31]. Moulds and yeasts (especially *Saccharomycotina*), also classified as members in the fungus kingdom, have always been part of the local folk knowledge, especially in connection with food [32].

When it came to the fruit bodies of the macrofungi, the peasantry was more indifferent. They observed that cattle eagerly ate boletes when grazing in the forests and named these boletes collectively *kosvamp* and *kosopp* (‘cow fungus’, ‘cow mushroom’), a myconym recorded since the eighteenth century. According to some records, this fodder made the taste of milk unpleasant [33]. People who dislike mushrooms still use *kosvamp* as a derogatory name for mushrooms in general [2, 16, 34].

In southernmost Sweden, the peasantry knew of larger mushrooms which they called *puggehattar* (‘toad’s hats’). These were considered to be not only inedible but also dangerous to eat [35].

### Present situation

However, attitudes changed during the twentieth century, and nowadays, urban people, especially of the middle class, are picking mushrooms for consumption and as a pastime [2, 16]. Commercial harvesting of wild mushrooms for domestic consumption and export also exists. Edible fungi have become increasingly popular non-timber products of the northern forests. Mushroom picking provides an important source of extra income for immigrants, especially Thai women, and foreign seasonal workers [1].

**Fig. 1:**
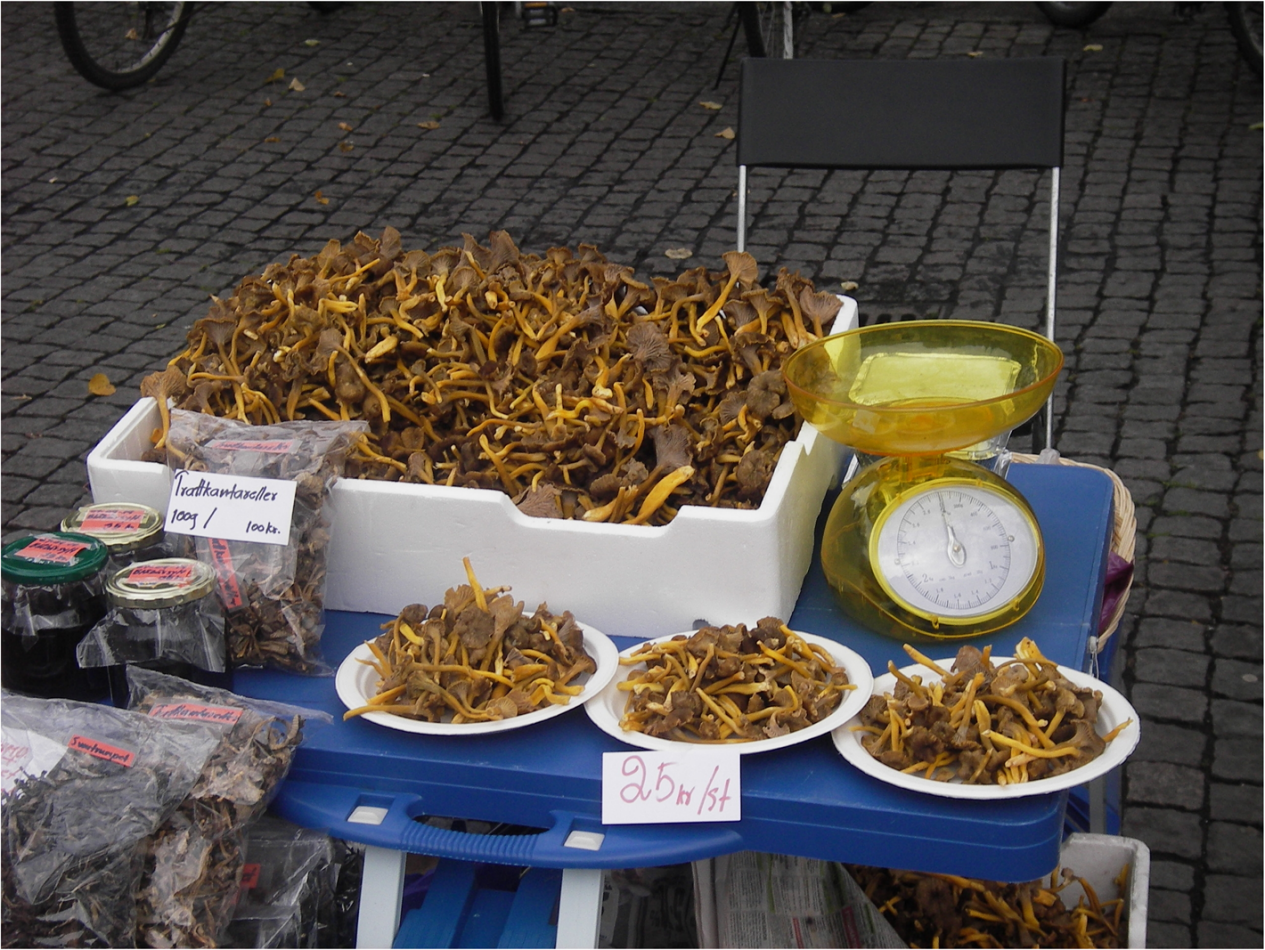
*Craterellus tubaeformis* for sale at a Thai market stall on Vaksala torg, Uppsala (photo: Navarana Ingvarsdóttir, 2008)

Mushrooms are available in weekly farmer’s markets during season (Fig. 1). There is in contrary to some other European countries no restriction in Swedish law against selling unprotected forest products, including mushrooms [36]. However, there are general EU rules on foodstuffs, which imply, inter alia, a risk assessment before marketing new foods. Such a risk assessment has been done on behalf of the Nordic Council of Ministers for foodstuffs that the expert group considered suitable for traded mushrooms. However, guidelines from the Swedish Food Agency for mushroom species suitable for marketing are available [37].

Within the last few decades, the field of ethnobiology has grown, transformed itself, and diversified [38]. Also contemporary urbanized population relates to and uses the biota in various and complex ways. While many contemporary ethnobiologists take a stand in a devolutionary premise to save as much of what they regard as ‘traditional knowledge’ before it disappear, we must widen our scope to study contemporary, modern uses of the biota. With 88% of Sweden’s population living in urban areas [39], the gathering activities among city dwellers have become an interesting field of research [40, 41].

Ethnomycological studies have until recently been very rarely conducted in this part of the world [42]. However, this has changed in the last few years and elsewhere in Europe, particularly in Poland, an increasing interest can be observed [40, 43, 44]. The northern part of Europe, especially Scandinavia, has traditionally been a mycophobic stronghold. Sweden is no exception [1, 16]. Now, this is fast-changing, and as a country rich in edible fungi resources, the interest here in making use of them is thriving. In 1980, ecologist Lars Kardell estimated that 3600 million litres of mushrooms (and 1000 million litres of berries) were growing on Swedish forestland every year. In addition, only a fraction of the mushrooms growing in Swedish forests every year is actually harvested [45]. In 1977, 21.8 million litres of mushrooms were harvested in the forests. A survey in 1997 showed that around 40% of the urban population harvested mushrooms. In 1995, Swedish households gathered altogether 14.4 million litres of mushroom for their own consumption [46].

## Purpose

The general objective of this article is to highlight the contemporary use of wild harvested mushrooms against a historical background and to discuss changes in the attitudes towards edible mushrooms over time. Thanks to earlier ethnological research and recorded data, for instance Keyland [10] and Egardt [2], we can provide not only a synchronic perspective but also a diachronic perspective indicating the development of interest in edible mushrooms in the twentieth century [47].

## The cultural, environment, and social setting

Contemporary Sweden covers an area of approximately 450,295 km^2^. Together with the islands of Gotland and Öland in the Baltic Sea, it forms the eastern part of the Scandinavian Peninsula. The climate is temperate with nearly 60% of the country being covered with forests and 15% located north of the Arctic Circle. Natural vegetation varies considerably due to the various climatic zones and ecological settings with mountains, forest regions, coastal areas, and agricultural landscape. Eight vegetation zones can be distinguished in Sweden. The boreal zone and its sub-zones cover the largest part of the country [48].

In the eighteenth century, Sweden was still poor, and despite considerable effort, the provision of sufficient foodstuffs for the population was far from reliably secured. In some parts of the country, the peasantry experienced frequent crop failures, so food crises and famine were a constant threat [49–51]. Agriculture remained primitive. In the subsistence economy, wild plants were essential for construction, hygienic and technical purposes, handicraft, dyes, animal fodder, and remedies, although seldom for human nutrition [19].

During the nineteenth century land reforms, ditching projects, modernization of agriculture and cattle breeding, better transportation through steamboats and railways, and better health care that resulted in the epidemiological transition improved the living situation of the population. Sweden became industrialized rather late, and in the 1880s, a large part of the population was still rural and poor [51, 52].

Although considered linguistically homogenous, modern Sweden still holds a cultural diversity of many groups speaking various minority languages. Traditional minorities include the indigenous Sámi, divided in various dialects and groups, who have a background in a full or semi-nomadic life style. Due to the long historical unification with Finland (until 1809), various Finnish-speaking groups still exist within the contemporary borders [53].

In the year 1700, Sweden’s population was only 1.4 million. By 1900, it had reached 5.1 million people. The country has remained sparsely populated, especially in the north [51]. Contemporary Sweden has a fast-growing population, consisting of almost 10.1 million inhabitants (2017), mainly due to immigration of refugees. The greatest part of the population lives in urban areas, and the education level is high. In the year 2000, 32% of the Swedes held a tertiary degree, placing Sweden as the fifth country in the Organisation for Economic Co-operation and Development (OECD) in that category. The immigrants are estimated at around 15% (1.3 million foreign born) of the population and originate from most countries of the world. In 2016, the largest foreign-born immigrant populations in Sweden originated from Finland, Syria, Iraq, Poland, Iran, former Yugoslavia, Somalia, Bosnia-Herzegovina, Germany, and Turkey [54].

Although export of timber products and iron ore has always been an important part of the economy, the agricultural sector has fallen to only 2% in recent years. In the 1870s, approximately 70% of Sweden’s population lived in the countryside. Nowadays, 85.4% (2017) reside in urban areas. In addition, intergenerational social mobility has been high. As a result, the generations growing up in the post-World War II era with working-class backgrounds have received higher education compared to their parents. Sweden is now a post-industrial society with a majority of the population living in the largest cities of the country [54].

Beside ecosystem services and timber products, forests provide numerous other economic, environmental, and social benefits for the Swedes. The forests and woodlands are important for recreation as well as for non-timber products. The Swedish population, although very urbanized, still has a close relationship with the forests. Many people spend time in forests just for recreation and meditation; others are hunting or gathering. Hunting, especially for elk, *Alces alces* (L., 1758); roe deer, *Capreolus capreolus* (L., 1758); and wild boar, *Sus scrofa* L. 1758, is a popular pastime for many Swedes [27]. There are over 300,000 licensed hunters, 7% of whom are women [46, 55]. Although picking berries for household consumption has decreased in the last decades, they are still of importance for commercial harvesting. The economically most significant and most popular wild berry species in Sweden are cowberry, *Vaccinium vitis-idaea* L.; bilberry, *Vaccinium myrtillus* L.; cloudberry, *Rubus chamaemorus* L.; and raspberry, *Rubus idaeus* L. They are primarily forest and mire species inhabiting various forests and peat lands [1, 36, 56]. The reindeer lichen species *Cladonia alpestris* (Opiz) Pouzar & Vezda form extensive carpets in the driest woodlands of the northern part of the boreal coniferous zone. The lichen is used for ornamental purposes, mostly exported to central Europe, where the leading importers are Germany, Austria, and Italy [57].

## Methods

The human-biota relationship is complex, and therefore, ethnobiologists have to use a variety of methods when collecting data [58–60].

The usage of qualitative questionnaires, originally an ethnological method for documenting and collecting material about everyday life, is a fruitful method for gathering information about mushroom hunting and consumption. The responses to qualitative questionnaires consist of memories, opinions, and experiences and thus offer understandings of great value [61].

Throughout the twentieth century, questionnaires have been used several times in order to document Swedish attitudes towards and utilization of mushrooms. The Folklife Archives at Lund University distributed two questionnaires in 1950: LUF 72 ‘Plants in household, medicine and magic’ (‘Växter i hushåll, läkekonst och magi’) and LUF 74 ‘Food stuff and prejudices’ (‘Födoämnen och fördomar’), which have been analysed by ethnologist Brita Egardt [2]. Another survey was conducted by the Swedish Gallup Institute of housewives in the 1950s, which included a few questions about the use and preservation of mushrooms within Swedish households [62]. In 2010, a questionnaire ‘The nature for me’ (‘Naturen för mig’) was distributed jointly by the folklore archives in Sweden. Gathering activities have been rather superficially analysed by ethnologist Bengt Edqvist [63]. The results from the surveys mentioned above have all been used when conducting this study.

**Fig. 2:**
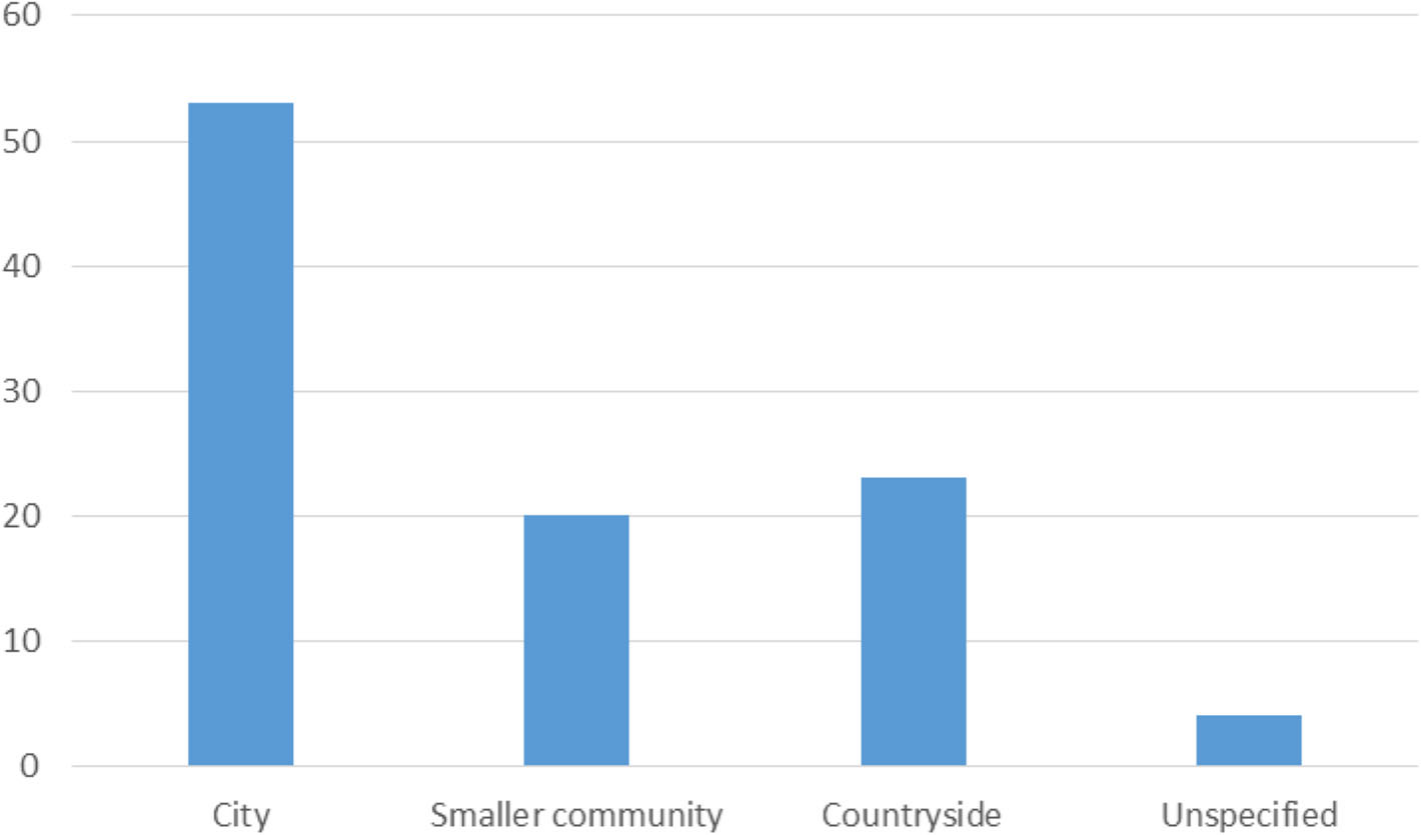
Amount of study participants originating from city, smaller community, countryside, and unspecified environments

In addition, a new open-ended questionnaire was constructed and dispersed. Throughout the limited time-period of October to November 2017, a total of 100 questionnaire responses were analysed. The group of survey participants comprised 56 women and 44 men. A majority of the respondents lived in Uppsala, the fourth largest city in Sweden with 219,000 inhabitants in 2017 and the oldest centre of higher education in Scandinavia, Uppsala University. As a result, many of the study participants ended up being academics themselves, some even mycologists. Consequently, the results are most likely to have been affected by this particular group of respondents. However, even though most of our participants live in Uppsala, they still have various geographical background with a few of them being born in other countries than Sweden (Estonia, Finland, Poland, and USA). A slim majority, 53 of the respondents, grew up within an urban setting, while 23 originate from smaller communities and 20 come from the countryside. Four of the questionnaire respondents did not specify their upbringing environments (Fig. 2).

Further empirical data for the contemporary perspective was collected through participatory and non-participatory observations, informal interviews with vendors and customers at weekly markets, and with consumers of mushrooms in various situations [64].

Observations among and discussions with sellers and consumers have been conducted at Uppsala’s two main open-air markets (Fyris torg, Vaksala torg) on a weekly basis from July to December throughout the last 20 years [1]. Data has also been obtained from newspaper reports and social media platforms describing and discussing Sweden’s contemporary consumption of mushrooms. Since mushroom hunting has become extensively common among the Swedish middle class, the authors have both taken part in many mushroom gathering activities since childhood. The senior author (IS) has observed, harvested, and consumed mushrooms since the 1950s, while the junior author (HL) holds a more limited experience of mushroom gathering consisting of the time period since the early 2000s. Finally, large numbers of published handbooks, cookbooks, and recipe collections have also been consulted for this study [65, 66].

## Results and discussion

### Slowly changing food habits

#### Pre-industrial times

Recipes in cookbooks from 1650 and 1664, respectively, confirm that some nobilities, influenced by French habits, were eating fungi (probably milkcaps, *Lactarius deterrimus* Gröger) during the seventeenth century [16]. A handbook for young noble men published in 1690 gives recipes of morels, champignons (*Agaricus bisporus* (J.E. Lange) Imbach), and *Calocybe gambosa* (Fr.) Donk**.** Morels (*Gyromitra esculenta* (Pers.) Fr.) are also mentioned in the kitchen accounts of the Royal court of Sweden (*hovförtäringen*) in the 1680s [16, 66]. In the eighteenth century, the cuisine of the tiny urbanized and internationalized upper class in castles and manors became more influenced by French cuisine. They came in contact with mushrooms also through their chefs, which were originating from southwestern Europe. Therefore, there was a market for products that were not used among the peasantry. For instance, in August 1741, Carl Linnaeus observed, in his home province Småland, how young peasant women were selling morels: ‘We saw farm girls collecting morels in the woods; they [i.e. the morels were big and beautiful and one pottle of morels was sold for four styver,’ [67].

In addition, recipes with morels are also mentioned in the most famous cookbook of the 1700s [68] and chanterelles were stated as food in the end of the eighteenth century [69]. Some attempts were made to promote mushroom export in the 1820s, but no species are mentioned in the sources [70]. With the exception of the interest in morels among the French-influenced upper class, fungi was otherwise non-existent in the food culture for another century or two [2, 16, 66].

From the 1830s until today, there has been extensive propaganda for the use of mushrooms as human food [9]. In the nineteenth century, we encounter names such as Elias Fries, Nils Johan Andersson, and Johan Wilhelm Smitt as authors of brochures intended to promote consumption of fungi in Sweden [71–73]. Elias Fries (1794–1878) was a pioneering academic mycologist and professor in practical economy. In 1836, he published a book with many examples of how mushrooms were being used in the cuisine of many other countries. He noted that Sweden had large resources in fungi which were not exploited and the Swedish people should be taught to use them [74]. Ethnologist Egardt emphasized that the propaganda for edible fungi as a foodstuff must be seen against the background of philanthropic and economical trends, typical of the time [2]. In particular, it was relevant to find nutrients that the peasant population could use as a substitute for harvest loss and famine years [9]. The primary objective of the mushroom propaganda was first and foremost to enlighten the peasantry about suitable emergency food in time of need [75].

During the period of food shortage and famine in the 1860s, economic societies, governors, and local provincial governments made many attempts to promote new food items among the peasantry, for instance lichens and fungi. The demonstrators gathered peasants in the villages to taste dishes prepared from several kinds of fungi, usually in the form of creamed mushrooms, mushroom soup, and mushroom bread. However, the peasants usually feared the fungi and no one dared to taste the dishes before [9, 49] the demonstrators had eaten them, and even then, it was difficult to persuade most people. An interesting exception of locals who tasted the dishes was Våmhus parish in Dalecarlia in the autumn of 1867. Many parishioners in Våmhus were part of an itinerant working force that periodically made its way to Russia and who therefore had heard about and even eaten mushrooms. However, they still did not incorporate mushrooms into their local cuisine [9].

The starving peasantry was not convinced. The use of mushrooms and other surrogates, such as lichens and other organisms available in the landscape, was a method of increasing accessibility to food through the use of the labour of one’s own household. Food could be found in the nature outside the realm of normal production through the use of available free labour (children, the elderly) among the peasants. In many areas, mushrooms were regarded as being merely cattle fodder. The peasants had also observed that they contained larvae and insects. The use of mushrooms as fodder certainly made the propaganda for their acceptance difficult. Edible fungi and other new food items met neither the criterion of cultural acceptability nor human dignity [49, 75].

There are some records from the early nineteenth century that are describing that Russian prisoners of war located in Sweden picked mushrooms. This phenomenon caused wonder and disgust among the locals who observed it [76]. Also, itinerant Kalderash Roma, who moved to Sweden from Southeastern Europe in the late nineteenth century, gathered fungi, mostly chanterelle (*C. cibarius*) and Boletales [77].

Even though the demonstrators did not successfully reach the peasants with their mushroom propaganda, they still became interested in fungi as an important foodstuff for themselves. Consequently, several handbooks were published during the second half of the nineteenth century.

**Fig. 3:**
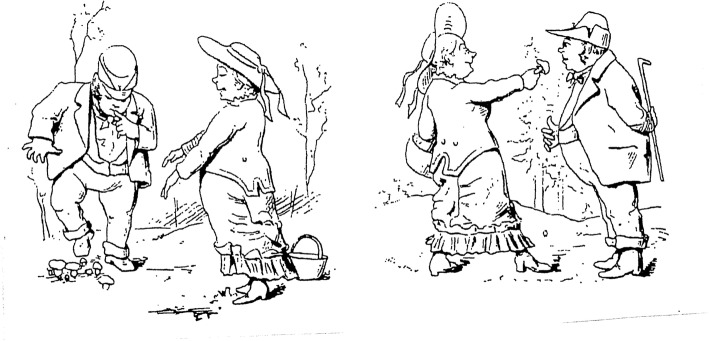
The newly awakened interest in picking mushrooms for food among the 1880s bourgeoisie became a subject for caricatures (from *Krumelurer* 1881)

During the 1880s, the urban gentry began to accept mushrooms. It was a kind of vogue to gather and consume mushrooms. About the same time, associations, such as Stockholm’s svampvänner (‘Stockholm’s Friends of Mushrooms’), were founded to provide information about edible mushrooms (Fig. 3). As a result, among the majority of the population, during the beginning of the twentieth century, fungi were considered as being upper-class food [2]. In the early twentieth century, it was still only the people of the gentry and townspeople who consumed mushrooms [35, 66].

Still, the twentieth century was the time when mushrooms begun to be accepted as food in wider circles [78]. Through the folklife records, we can obtain information regarding when fungi began to be consumed in the Swedish countryside. Mushrooms were unknown as a food ingredient until about 1912, according to an informant from Markaryd parish in Småland [79].

Therefore, educational efforts were important in order to teach the population on how to use these neglected resources. In the 1920s and 1930s, educational posters were used in primary schools to promote the population’s interest in edible fungi [80]. For a long time, however, Swedes only consumed chanterelles and *Boletus edulis* Bull. [2, 78]. Several handbooks were published in large editions, which at least reflect an interest for edible fungi [66, 68]. These handbooks have also been of great importance to promote myconyms—usually neologisms invented by Swedish mycologists which have been accepted by mushroomers [16] (Table 1).

**Table 1:** Published books and pamphlets for identification of fungi species, including recipes

1836–1899 23 titles	
1900–1909 6 titles	
1910–1919 11 titles	
1920–1929 4 titles	
1930–1939 14 titles	
1940–1949 28 titles	
1950–1959 23 titles	
1960–1969 8 titles	
1970–1979 32 titles	
1980–1989 66 titles	
1990–1999 47 titles	
2000–2009 38 titles	
2010–2015 16 titles	

Source: libris.kb.se, accessed 1 January 2018

#### Post-war era

**Fig. 4:**
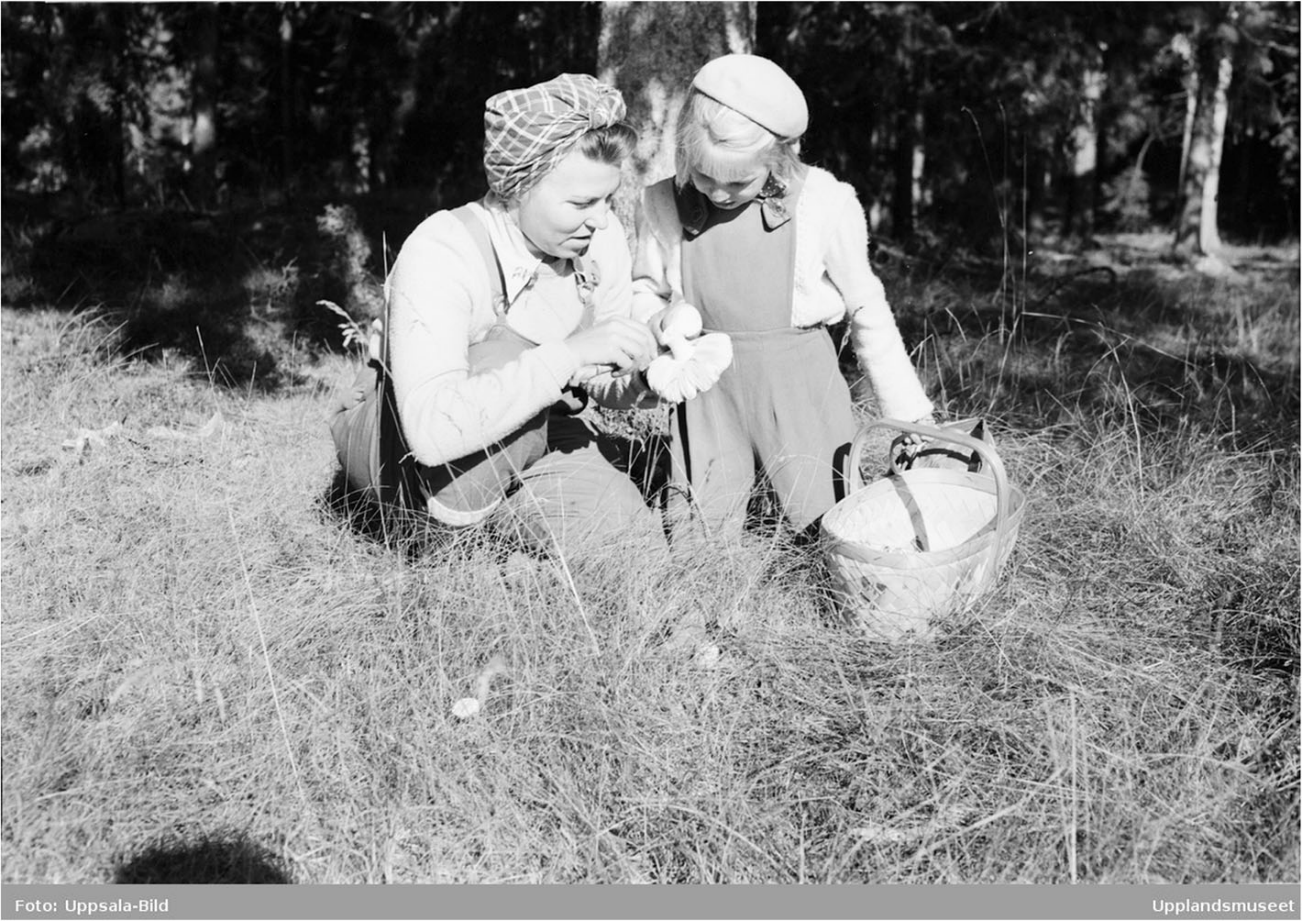
Mushrooming in the vicinity of Uppsala in 1950 (photo: Uppsala-Bild, courtesy Upplandsmuseet, Uppsala)

**Fig. 5:**
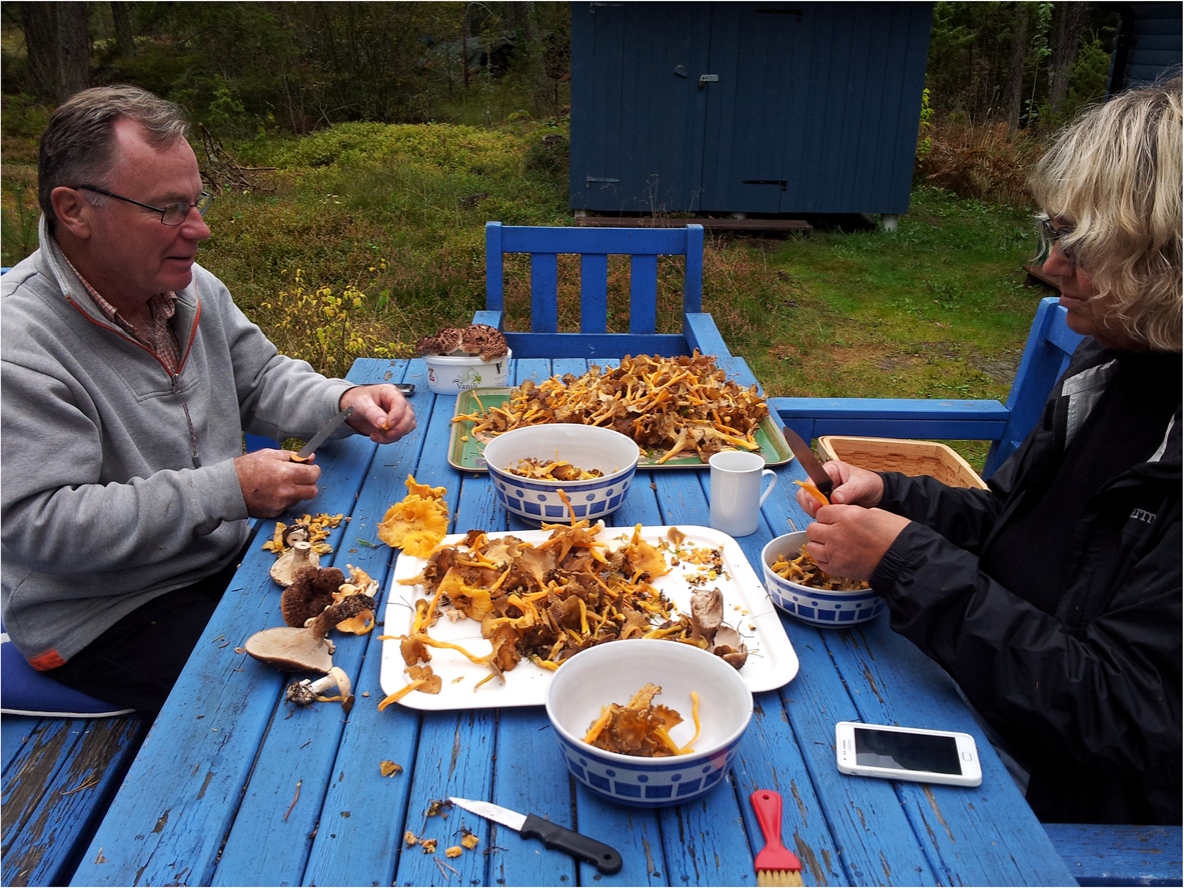
Hasse and Kerstin are cleaning the harvested mushrooms with small knives and brushes (photo: Birgitta Bjärkstedt, 17 October 2017)

**Fig. 6:**
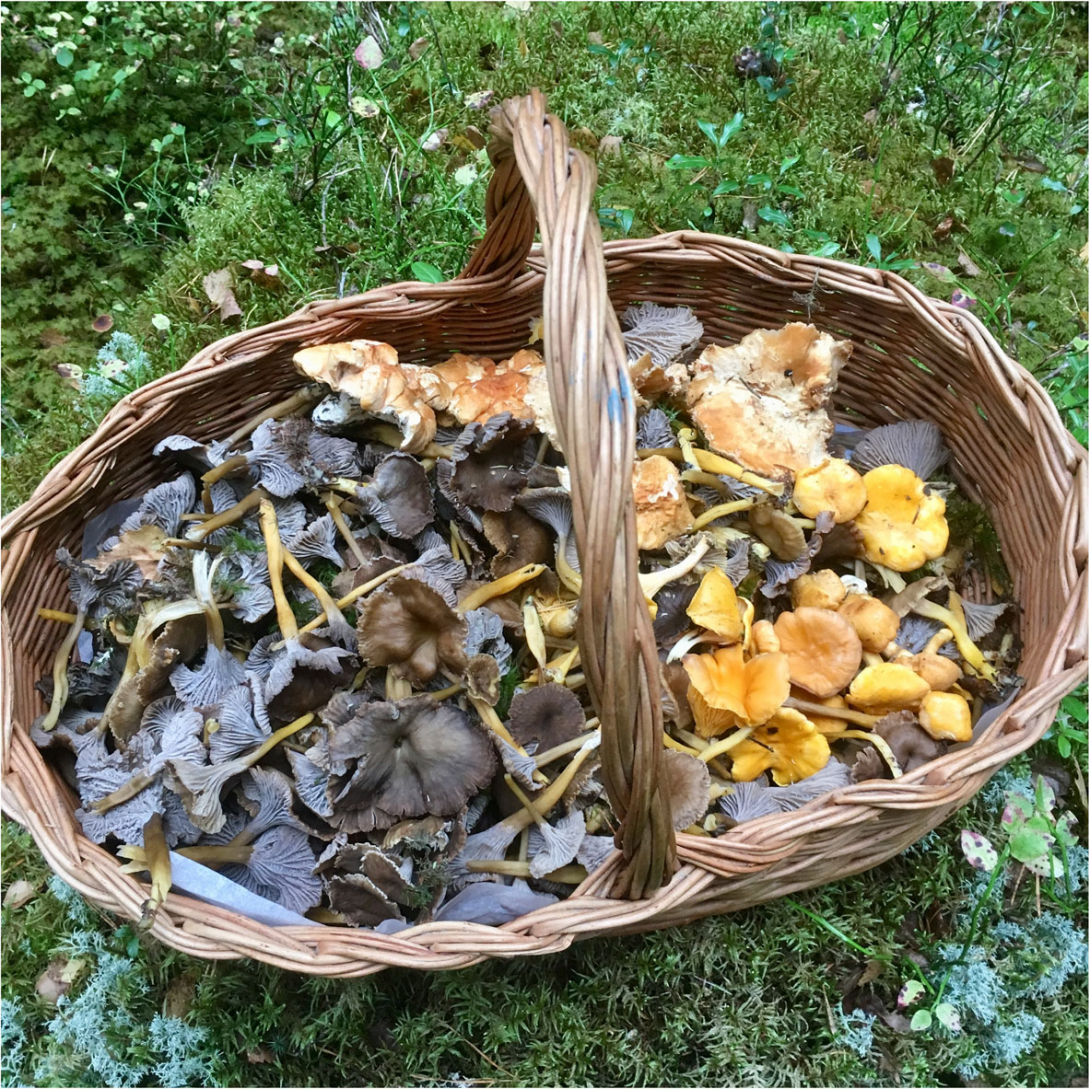
The basket is a typical part of the necessary equipments for the mushroom picker (photo: Simon Sorgenfrei, 7 October 2017)

In the process of accepting fungi as food, we are currently witnessing the final phase of a taste change, ethnologist Brita Egardt concluded in her research [2]. This coincides with the general development of Sweden as an urban welfare society. The change that was occurring during the post-World War II period has been described as ‘the record years’ in Sweden. Between 1947 and 1974, the Swedish economy grew at an average rate of 12.5% annually. The urban population, living in towns of populations reaching over 15,000 people, grew from 38% of the total population in 1931 to 74% by 1973 [51]. Sustained by an export boom of automobiles, heavy machinery, electronics, ship building, and heavy weapons, the per capita income increased by as much as 2000%. Sweden had successfully moved into the high-income group of countries by 1955–1956. During the same time period, the urban people of Sweden developed an interest for the nature, which became accessible for town dwellers with private cars. To be able to stroll through and make use of the forests and their products had been of great importance for urban people during the post-war era [1, 46, 80] (Figs. 4, 5 and 6).

The interest in mushrooms has continuously been promoted by enthusiasts [1, 16]. Important for the mushroom propaganda in the post-World War II era in Sweden was Nils Suber (1890–1985), mycologist and author of several handbooks that was published in several editions. A disciple of Mr. Suber and famous contemporary mushroom expert and advocate, who often predicts the fungi season in media, is botanist Pelle Holmberg (born 1948). He has made himself known as an important public educator in the field of mycology. He has written around twenty books about fungi. In lectures and media, he has praised mushrooms as food for decades. In the early 1970s, he met with Mr. Suber and collaborated with him in many lecture tours. Holmberg wrote course material for study circles. In the early 1980s, there were over 500 study circles per year in Sweden. They also initiated mycological societies in Sweden [81]. A central organization, Swedish Mycological Society, was founded in Femsjö parish (where mycologist Elias Fries was born) in the province of Småland in August 1979 [16].

Information efforts for gathering and eating mushrooms is ongoing, and every summer and autumn, newspapers and magazines are filled with tips about edible species and their use. There are plenty of mushroom guides, and for some decades, professional trained mushroom consultants (educated in Härnösand and later Umeå), who help the ignorant to determine edible species, have become increasingly requested [82, 83].

Many immigrants with East and Central European background are eager mushroom hunters, often with a rather good knowledge of various edible species. In the 1980s, IS encountered Russians, Czechs, and Poles in the forest while hunting for mushrooms [1, 83]. Harvesting mushrooms, with the exceptions of chanterelles and a few other species, was still a middle class concern. The vast majority of Swedes still harvested cowberries and bilberries in the forest [1, 36, 46].

One way to promote an interest in mushrooms has been to appoint province mushrooms, in the same way as province flowers, which is the idea of selecting a species to represent each province of Sweden. When it comes to flowers, the idea was brought to Sweden in 1908 by theologian Paul Peter Waldenström (1838–1917) and has been popular since, promoted by the school system, local heritage societies, and tourist industry. Most province flowers are important symbols and trademarks today [82]. Attempts to promote the idea of province birds, fish, insects, lichens, mammals, trees, etc. have been common during the last two to three decades but with limited success. Mycologists suggested a list of province mushrooms in the 1980s, all of them edible species such as *Sarcodon imbricatus* for Gästrikland, *Craterellus cornucopioides* (L.) Pers. for Södermanland, and *B. edulis* for Uppland, but this is hardly widely known among people although it is mentioned in handbooks, encyclopedias, and newspapers [84].

### Chernobyl disaster effect

A brief but still serious set-back in the interest for mushrooming was the Chernobyl disaster in the former Soviet Ukraine in April 1986. It was a catastrophic nuclear accident that also affected Sweden in many ways. Rain clouds tied radioactive particles that drifted with the winds and caused radioactive deposition over wide areas. In a few days, the deposition area had spread to Sweden, with certain parts of central Sweden becoming especially contaminated. Dangerous radioactive isotopes were absorbed by, among other things, fungi in the forests. Ingestion of such mushrooms was therefore inappropriate before the radioactivity decreased [85].

As a result, many Swedes refrained from picking mushrooms in the following years. Still some people refer to the Chernobyl disaster as a factor affecting their mushroom picking habits.

A male survey participant, born in the 1940s, reported the following:

…I had a few good mushroom picking sites a couple of miles outside of Uppsala, but after Chernobyl we stopped gathering there. Actually, we stopped picking mushrooms entirely, from the region surrounding Uppsala…

A woman, born in the 1970s, stated:

…I picked a lot of mushroom together with my parents until Chernobyl. Then we stopped because of the fungi’s high degree of cesium [… My dad used to say; “Now people are going out to collect Becquerel”…

Still in 2017, as much as ten percent of the wild boars that are getting killed by hunters in some parts of the Swedish provinces of Gästrikland, Uppland, and Västmanland had too high cecium-137 levels to be suitable as food, due to their high consumption of contaminated fungi [86].

### Twenty-first century: food and pastime activity

In this section, mainly, the results gained through the questionnaire survey will be presented in combination with long-term observations of the authors as well as information on gathering and consumption of fungi documented through Facebook and Instagram, two social media platforms with great popularity in Sweden during the sampling period.

These days, autumn means fungi season for many urban people in contemporary post-industrialized Sweden. Mushroom hunting is a rather popular leisure activity during the months of August to November [1, 87]. Already in June to July *Cantharellus pallens* Pilát (=*C. cibarius* Fr.) can be harvested in deciduous forests in the Stockholm-Mälaren region.^2^ Thanks to the so-called Everyman’s rights, allowing Swedes and foreigners to roam, hike, and gather berries and mushrooms not only on public but also on private land, these resources are available both for private consumption and for commercial harvesting [80, 87]. It is also possible to pick mushrooms in urban parks and lawns. In Uppsala, the authors have observed people harvesting *Marasmius oreades* (Bolton) Fr., which is usually growing in fairy rings, early mornings in park lawns [1]*.*

We can observe the same development in neighbouring Nordic countries. In Finland, the traditions vary from the mycophilic Orthodox Finns in the east, influenced by its proximity to Russia, to the less enthusiastic west, taking its influences from Sweden [5, 14]. Nowadays, the situation is the same as in Sweden. Thanks to the official encouragement to collect edible fungi since the Second World War, access to forests and with the help of local mushroom advisors, people in Finland today have a great knowledge and interest in edible fungi [88]. In Denmark, mushrooming is still small-scale and infrequent local collections only, probably due to the lack of tradition and limited access to mushroom habitats [89]. Norway has about the same development as Sweden, and many people have discovered that picking mushroom for food and recreation is bringing pleasure. Also, the Norwegians enjoys the same right of access to, and passage through, uncultivated land, including foraging berries and mushrooms, in the countryside as the Swedes [6].

### Knowledge of edible species

In total, the 100 study participants mentioned 136 different vernacular names of edible mushrooms that they knew of and gathered currently or which they had collected during previous periods of their lives. In this study, we chose to present a list of mushroom taxa mentioned by at least three study participants, as being collected for their edible properties. Mushroom taxa mentioned by one or two study participants only (*n* = 61) were estimated to not work as representatives of fungi taxa consumed by the general Swedish mushroom foraging community, but to rather illustrate untypical uses by one or two people, sometimes influenced by a background involving living abroad or gaining mycological expertise through advanced mycological studies. Therefore, the authors chose not to present these answers in the final set of results (Table 2).

**Table 2:** List of mushroom taxa mentioned by at least 3 study participants, as being collected for their edible properties

Scientific name	Vernacular name	Times mentioned
*Cantharellus cibarius* Fr.	Gul kantarell, blek kantarell, sommarkantarell	98
*Boletus edulis* Bull.	Karl johan, stensopp	88
*Craterellus tubaeformis* (Fr.) Quél.	Trattkantarell, höstkantarell	73
*Craterellus cornucopioides* (L.) Pers.	svart trumpetsvamp	38
*Lactarius deterrimus* Gröger	Blodriska, granblodriska	34
*Agaricus* sp.	Champinjon	31
Boletales	Sopp	30
*Suillus luteus* (L.) Roussel	Smörsopp, smörsvamp	30
*Macrolepiota procera* (Scop.) Singer	Stolt fjällskivling	28
*Craterellus lutescens* (Fr.) Fr.	Rödgul trumpetsvamp, gul trumpetsvamp, brandgul trumpetsvamp, gul trattkantarell	28
*Albatrellus ovinus* (Schaeff.) Kotl. & Pouzar	Fårticka	25
*Hydnum* sp., *Sarcodon* sp., *Hydnellum* sp., *Phellodon* sp. (folk taxon)	Taggsvamp	22
*Russula* spp.	Kremla	21
*Hydnum repandum* L.	Blek taggsvamp, blekgul taggsvamp	21
*Gomphidius glutinosus* (Schaeff.) Fr.	Citrongul slemskivling, citronslemskivling, citronskivling	18
*Lycoperdon* sp., *Calvatia* sp. (folk taxon)	Röksvamp	15
*Morchella* sp., *Gyromitra* sp., *Helvella* sp. (folk taxon)	murkla	14
*Clavaria* sp., *Clavulina* sp., *Clavicorona* sp., *Clavulinopsis* sp., *Macrotyphula* sp., *Ramaria* sp., *Ramariopsis* sp. (folk taxon)	Fingersvamp	14
*Cortinarius caperatus* (Pers.) Fr.	Rynkad tofsskivling, rimskivling	14
*Leccinum aurantiacum* (Bull.) Gray	Aspsopp, eksopp	14
*Craterellus* sp.	Trumpetsvamp	12
*Sparassis crispa* (Wulfen) Fr.	Blomkålssvamp	11
*Lactarius* sp.	Riska	11
*Leccinum scabrum* (Bull.) Gray	Björksopp	11
*Agaricus campestris* L.	Ängschampinjon	9
*Sarcodon imbricatus* (L.) P. Karst.	Fjällig taggsvamp	8
*Suillus variegatus* (Sw.) Richon & Roze	Sandsopp, sandsvamp	7
*Lactarius deliciosus* (L.) Gray	Tallblodriska, läcker riska	7
*Lactarius volemus* (Fr.) Fr.	Mandelriska	6
*Coprinus comatus* (O.F. Müll.) Pers.	Fjällig bläcksvamp	6
*Armillaria mellea* (Vahl) P. Kumm.	Honungsskivling	6
*Russula integra* (L.) Fr.	Mandelkremla	6
*Leccinum versipelle* (Fr. & Hök) Snell	Tegelsopp, tegelröd björksopp	6
*Agaricus arvensis* Schaeff.	Snöbollschampinjon	5
*Hygrophorus camarophyllus* (Alb. & Schwein.) Dumée, Grandjean & Maire	Sotbrun vaxskivling, Sotvaxskivling, sotvaxing	5
*Lactarius torminosus* (Schaeff.) Gray	Skäggriska	5
*Marasmius oreades* (Bolton) Fr.	Nejlikbroskskivling	5
*Clitocybe nebularis* (Batsch) P. Kumm.	Pudrad trattskivling	4
*Boletus pinophilus* Pilát & Dermek	Rödbrun stensopp	4
*Gyromitra esculenta* (Pers.) Fr.	Stenmurkla	4
*Coprinus* sp.	Bläcksvamp	4
*Russula xerampelina* (Schaeff.) Fr.	Sillkremla	4
*Hydnum albidum* Peck	Vit taggsvamp	3
*Albatrellus confluens* (Alb. & Schwein.) Kotl. & Pouzar	Brödticka	3
*Hygrocybe* sp., *Hygrophorus* sp. (folk taxon)	Vaxskivling	3
*Hydnum rufescens* Pers.	Rödgul taggsvamp	3
*Morchella esculenta* (L.) Pers.	Toppmurkla	3
*Lepista nuda* (Bull.) Cooke	Blåmusseron	3
*Lactarius trivialis* (Fr.) Fr.	Skogsriska	3
*Imleria badia* (Fr.) Vizzini	Brunsopp	3
*Boletus reticulatus* Schaeff.	Finluden stensopp	3
*Russula decolorans* (Fr.) Fr.	Tegelkremla	3
*Ramaria flava* (Schaeff.) Quél.	Stor gul fingersvamp, gul fingersvamp	3

Scientific names obtained from Index Fungorum

Many of the vernacular terms ended up being synonyms, such as ‘*citrongul slemskivling*’, ‘*citronslemskivling*’, and ‘*citronskivling*’. In total, the 136 reported vernacular names represented 91 scientific species, 11 genera, and 2 orders. Furthermore, 10 of the mentioned terms exemplified folk taxa without scientific equivalents. One example of a reoccurring folk taxon was ‘*taggsvamp*’, which describes any mushroom species within the genera *Hydnum* sp., *Sarcodon* sp., *Hydnellum* sp., and *Phellodon* sp. Foremost, the label *taggsvamp* denominates fungi with prickles (*tagg* in Swedish) at the section of their basidium and with a tough consistency.

Out of the 136 mentioned vernacular names, 43 names were only mentioned by 1 participant once. This shows a great knowledge variety among the informants. On an average, the study participants reported 9 different mushroom species each. According to the questionnaire results, the pre-eminently most popular fungi species among the participants were *C. cibarius* (*n* = 98), *B. edulis* (*n* = 88), and *Craterellus tubaeformis* (*n* = 73).

### Mushrooms in contemporary Swedish cuisine

**Fig. 7:**
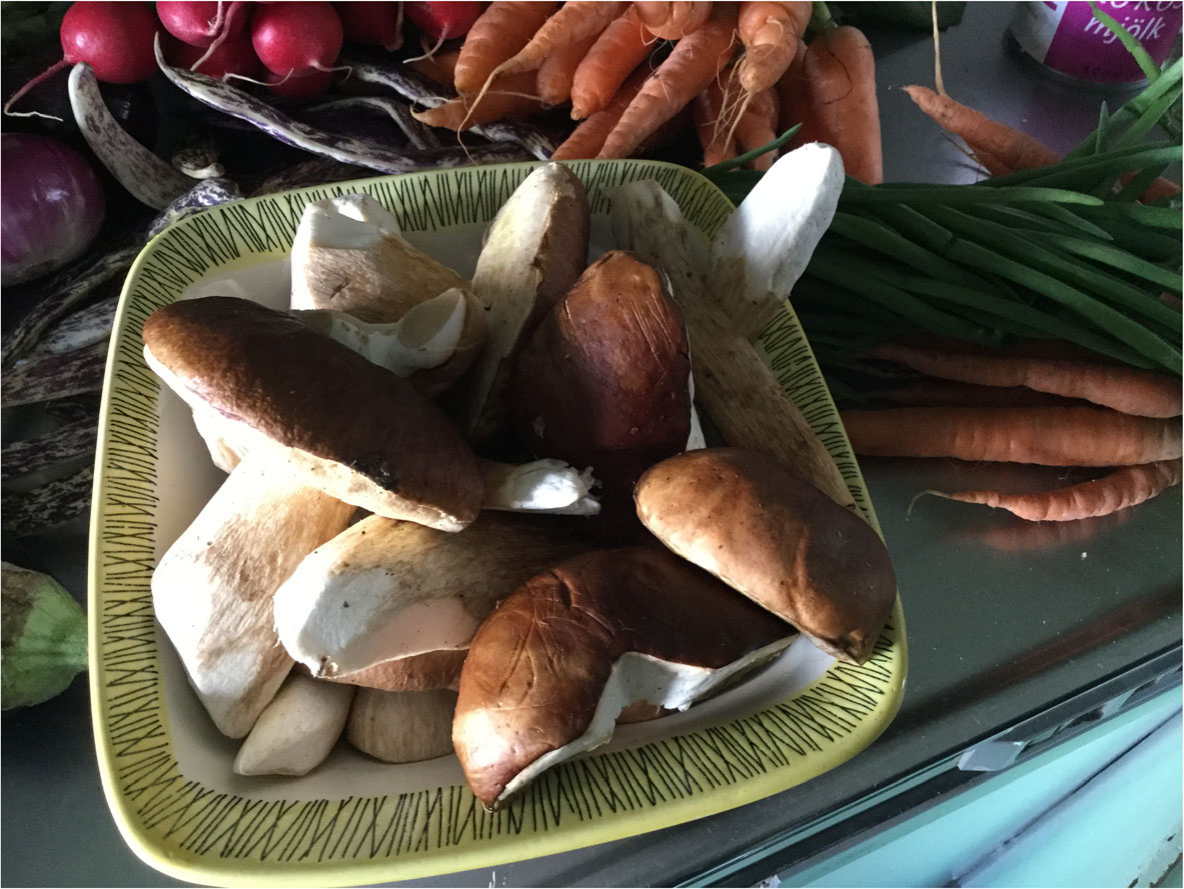
A nice harvest of *Boletus edulis* ready for the kitchen (photo: Ingvar Svanberg, 26 August 2017)

Nowadays, mushrooms play an important role in the Swedish cuisine and offer a wide variety of options in contemporary cooking [16, 66]. Many households harvest mushrooms for their own needs. Also, the food industry and restaurants are asking for fungi [78, 90]. Mushrooms have been considered nutritionally poor and rather a taste experience than being a real nutritional supplement. In recent years, however, researchers have noted that the fungi develop vitamin D and that the bioavailability of this vitamin from mushrooms is good in the human body [91] (Fig. 7).

As mentioned previously, the most commonly gathered mushroom according to the 2017 questionnaire is *C. cibarius*. It is also ranked among the tastiest species. Larvae do not infest it (in contrast to *B. edulis*, another popular species), and you can usually gather a larger number. Other popular species are *C. lutescens*, *C. cornucopioides*, and *C. tubaeformis*. The latter can be gathered as late is in November, and an industrious picker can gather a large amount of it. It is also being easily preserved by methods of drying [1, 66].

A large number of mushroom species are available in the general grocery stores: *Agaricus bisporus*, which is widely cultivated in Sweden and available as white champignon, chestnut mushroom and as portobello, shiitake (*Lentinula edodes* (Berk.) Pegler), and oyster mushroom (*Pleurotus ostreatus* (Jacq.) Quél.), but also (during autumn) wild species, such as *C. cibarius* and *C. tubaeformis*. The latter is also available as dried. Some of the informants answered that if they would eat fungi, they would only consume cultivated champignons, usually canned ones.

To fry chanterelles in butter, served on toast or Swedish crisp bread, is still the most appreciated fungi dish, based upon survey results, interviews, and media reports. Another popular dish, involving mushrooms, is stew with slices of reindeer meat and *C. lutescens.*

Some survey informants described how they preserved fungi in the past. A woman born in the 1940s reported:

…My parents ate fungi…They preserved chanterelles in cans after preboiling them. During the war years, they used taggsvamp (*Hydnum sp.*) and fårticka (*Albatrellus ovata*) in something that looked like pickled herring. Äggsvamp (*Bovista sp.*), they fried in large slices like pancakes…

A man born in the 1950s stated:

My parents pickled mushrooms, but stopped when they bought a refrigerator in the 1960s…

### Identification methods and knowledge transmission

**Fig. 8:**
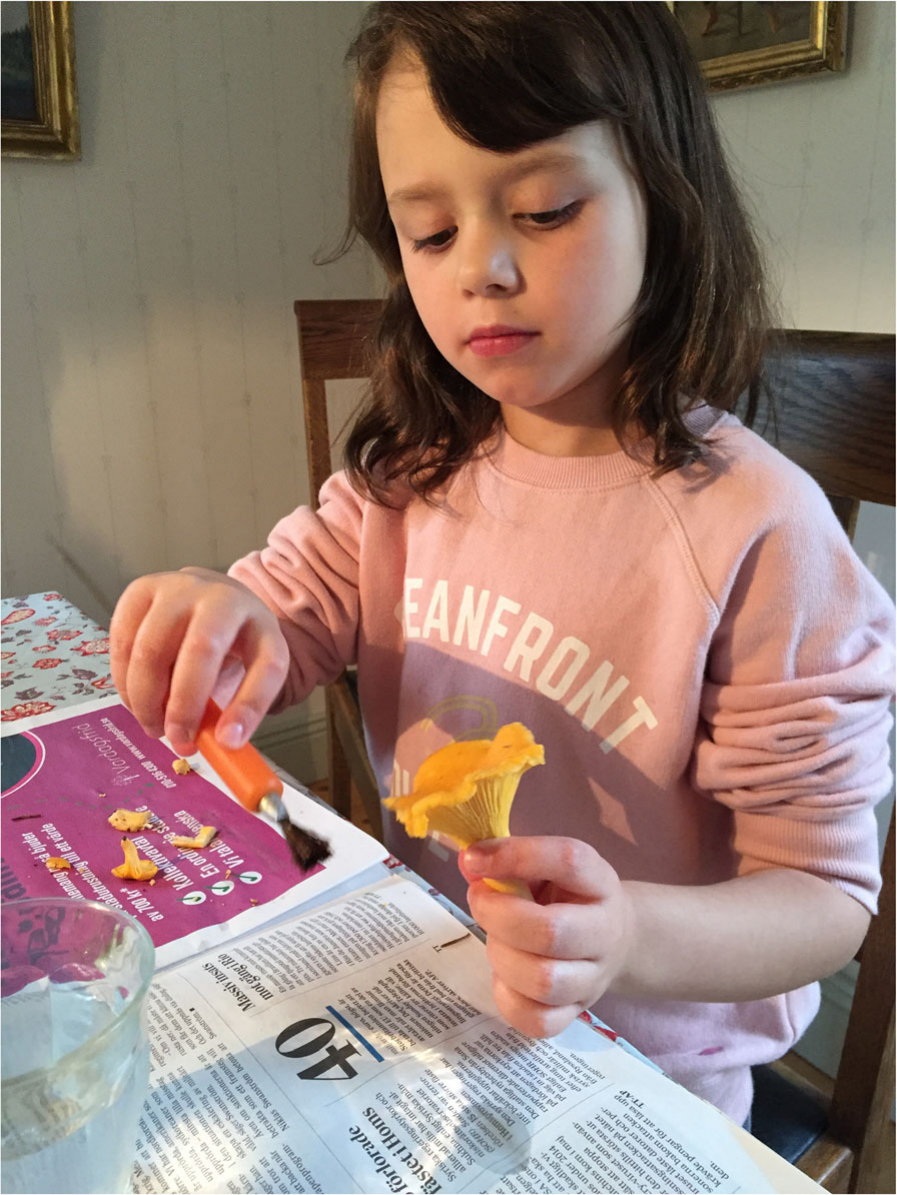
The yellow *Cantharellus cibarius* is the most popular species among mushroomers of all ages (photo: Mia Terent, 17 October 2017)

A majority of the study participants reported that they would only collect edible mushroom species that they were familiar with and completely certain of regarding their species identities. Some informants would gather the same variety of species as their parents did during their childhood (Fig. 8). In most cases, the transmitted information was said to be limited to a few taxa. And still, this quite narrow knowledge base was reported as being sufficient enough, sometimes leading up to an increasingly extensive knowledge in fungi as an adult.

One female informant, born in the 1940s, reported:

…We gathered a lot of fungi each year. My mum was an eager and good mushroom forager and knew about several species, many more than I can recall nowdays. She was also my first tutor in mushroom gathering…

Another woman, born in the 1960s, reported:

…My mother has always gathered fungi and she taught me about it as well. I gather the same species as she does…We often ate mushrooms when I lived with my parents and sometimes mum gathered fungi in the mornings that we then would consume well-fried on a sandwich before going to school…

One male informant, born in the 1950s, recalled that:

…My parents gathered mushrooms. In particular my father. He saw it as an almost spiritual experience to collect both fungi and berries. These activities were great contrasts compared to his profession as a construction worker...

Many participants reported that they did recognize a distinct difference between their parents’ and grandparents’ attitude towards mushroom foraging, with the grandparents being more reluctant towards mushroom consumption and the parents being curious and increasingly open-minded towards consuming new types of edible fungi.

One woman, born in the 1940s, reported that:

…I grew up with my grandparents. They did not eat fungi (or cow fungi as my grandfather called them), but my aunts and uncles ate mushrooms on the other hand. They made stews of chanterelles and put it on toast which they offered to me…

The old habit of using the derogatory term *kosvamp* (‘cow fungi’, ‘cow mushroom’) is still remembered and maintained by some. One female survey participant, born in the 1960s, verified this and did at the same time describe how she had experienced a difference between her grandparents regarding their interest in edible fungi due to their different professions.

My maternal grandmother and grandfather ate conserved chanterelles often, preferably as a complement to cod cooked in the oven. My paternal grandmother and grandfather, on the other hand, did not like to eat fungi at all [… I have interpreted this difference as a consequence of that my paternal grandparents were farmers and therefore viewed the mushrooms as food for their cows, while my maternal grandparents were fishers and owners of a fish seller store and thereby valued fungi in another way.

A large number of participants described how they learned about mushroom foraging as teenagers or adults, often through a friend or partner, as a consequence of their own parent’s disinterest in edible mushrooms during their younger years. Several informants claimed that their parents did not pick and consume mushrooms since they belonged to a mushroom-despising generation, which concerned most species (except for chanterelles) as possibly poisonous. Due to the lack of familiarity with mushroom picking, a number of participants reported that they themselves stayed hostile towards consuming wild fungi. One female participant, born in the 1970s, reported that she would never dare to gather edible fungi for consumption without the guidance of friends with extensive knowledge in mushroom picking.

Another woman, born in the 1980s, stated:

I love to spend time in nature and observe different mushrooms together with my children. I constantly think that I should learn more about fungi in order to teach my kids. My parents did not have any interest in mushroom picking. I would like to try to gather mushrooms. Although, I am scared of collecting poisonous fungi. Therefore, I do not regard myself knowledgeable enough to gather mushrooms. I feel that I should bring someone more experienced with fungi foraging along with me.

In other cases, the parental generation was knowledgeable and interested in foraging of fungi, but the knowledge transmission ended up being disrupted due to a lack of interest among the youth.

One woman born in the 1940s reported that:

…My parents were good at recognizing and gathering mushrooms. I remember foraging daytrips with lunch packs. Although, I do not think that I learned anything from it. I guess that I was relatively disinterested…

While a majority of the participants seemed to be reluctant towards collecting mushroom species, which they did not know perfectly well from beforehand, a relatively large group still reported that they tended to use handbooks for identifying mushrooms. Some stated that they found it amusing to identify, for them, unknown species, but that they did not have the courage to actually consume the ‘new’ mushroom taxa. A small group of the participants reported that they never gathered fungi, due to limited physical abilities or simply because of fear of gathering poisonous species by mistake.

### Poisonous mushroom

As mentioned in the previous section, some Swedes are still afraid of harvesting poisonous fungi by mistake. This is the case even though the number of reported intoxication cases in Sweden has been rare according to the statistics of the past years [92]. Nevertheless, handbooks and newspaper articles regularly warn the public about toxic fungi species. As a result, most people in Sweden know about the toxic species from the *Amanita* genus and the survey informants mentioned several other types of poisonous mushrooms in addition. Some of the participants were afraid of harvesting poisonous species by mistake to the degree that they prefer to avoid gathering mushrooms entirely.

For example, one female informant born in the early 1950s reported:

…We were very afraid of fungi! We avoided them as the Plague! I would never use mushrooms as an ingredient when cooking. They sell chanterelles on the square where I live, but I would never dare to buy them…

And even though the cases of intoxication are few, there are still poisonous mushrooms being harvested from the Swedish forests; some of them are a result of the actual handbooks. To rely on the information in field guides on edible fungi can be hazardous, especially if the book is not recently updated, since the information on the toxicity of a species can end up being obsolete [93]. One such example of a type of fungi, which has recently been reclassified from an edible species into a dangerous one, is *Gyromitra esculenta* (Pers. ex Pers.) Fr. [94]. It was long regarded a delicacy, mostly foraged by experienced mushroom gatherers, but has also been traded, both fresh and dried, requested by fine restaurants [37]. However, new research has shown that the recommended treatment of parboiling before preparation is not enough to make it free from the toxic substance gyromitrin. The consumption of one raw fungi specimen can cause death. Mushroom experts therefore suggest that *G. esculenta* should be replaced with a true morel, *Morchella conica* (L.) Pers. It does not need to be parboiled, but people do it anyway as a safety measurement [95]. Many of the survey informants described their, often complicated, relationship with morels.

A male informant born in the 1950s stated:

… My father had a certain repertoire of species that he gathered and regarded as edible. Morels [Gyromitra esculenta (Pers. ex Pers.) Fr.] for example. I do not gather these myself…

A female informant born in the 1960s reported:

…My parents collected morels […] and parboiled these for consumption. Since they told me that they were poisonous when we gathered them, but could not tell the degree of toxicity. They could not answer my questions about what part of the mushrooms that was dangerous. Then I refused to eat the fungi at all, even after preboiling. Today my parents have stopped consuming morels since they are not recommended to be consumed at all anymore…

And a woman born in the 1940s wrote:

…Due to new information about toxicity, I have stopped using many species that my parents consumed…

The Swedish Poison Information Centre reports that people who come to Sweden from abroad (immigrants, refugees, temporary visitors) are the most likely to suffer mushroom poisoning; Erik Lindeman of the Swedish Poison Information Centre told Swedish Radio News: ‘It’s common that those who are poisoned have a foreign background…It could be tourists, asylum seekers or migrants who go out in the Swedish forest and think they recognize mushrooms, which they know from their home countries’ [96].

The foreigners and immigrants are over-represented among those contacting the Swedish Poision Information Centre about the risk of have eating poisonous fungi. In 2017, a Syrian woman in Teckomatorp in southern Sweden became seriously ill after eating the poisonous destroying angel (*Amanita virosa* Fr. Bertill.). In order to combat these situations, the Swedish Migration Agency plans to disseminate warning leaflets about dangerous mushrooms that grow in Sweden to refugee accommodations. The brochures are also available on the Poison Information Centre’s website and have been translated into 29 different languages in order to be accessible for as many asylum seekers as possible [97].

One interesting aspect of all this is that as late as in the 1960s, there was an extended notion that one could determine if a mushroom species was toxic or not by tasting it. Several informants have testified that their parents used this method.

For example, one female informant born in the 1950s described the behaviour of her own mother:

…During the 1950s and 1960s my mother gathered mushrooms and separated poisonous mushrooms from good by tasting them…I only collect safe mushrooms and do not use my mom’s tasting method…

### Ticks and mushroom hunting

Mosquitoes (Culicidae) may be a nuisance when people are harvesting berries and picking mushrooms, although the mosquito season is usually over when it is time mushrooming. Repellents are readily available in pharmacies and stores for people strolling in forests. However, in our informal interviews, some people affirmed that they are scared off from mushroom picking by the profusion of castor bean tick, *Ixodes ricinus* L., in the forests.

When mushrooming, there is a risk for being bitten by the ticks, which thanks to increasing population of elks are very common. The risk of being bitten is rather high, if you are not properly dressed [98]. Since the tick can transmit both bacterial and viral pathogens such as Lyme disease (*Borrelia*) and tick-borne encephalitis (TBE), it is feared by many Swedes, especially along the east coast and in the vicinity of Lake Mälaren where Lyme disease and TBE is most common. As an experienced fungi forager, you are usually dressed to avoid tick bites and many individuals have taken vaccine against TBE, but there are no protection against Lyme disease [99].

### Mushroom spots

**Fig. 9:**
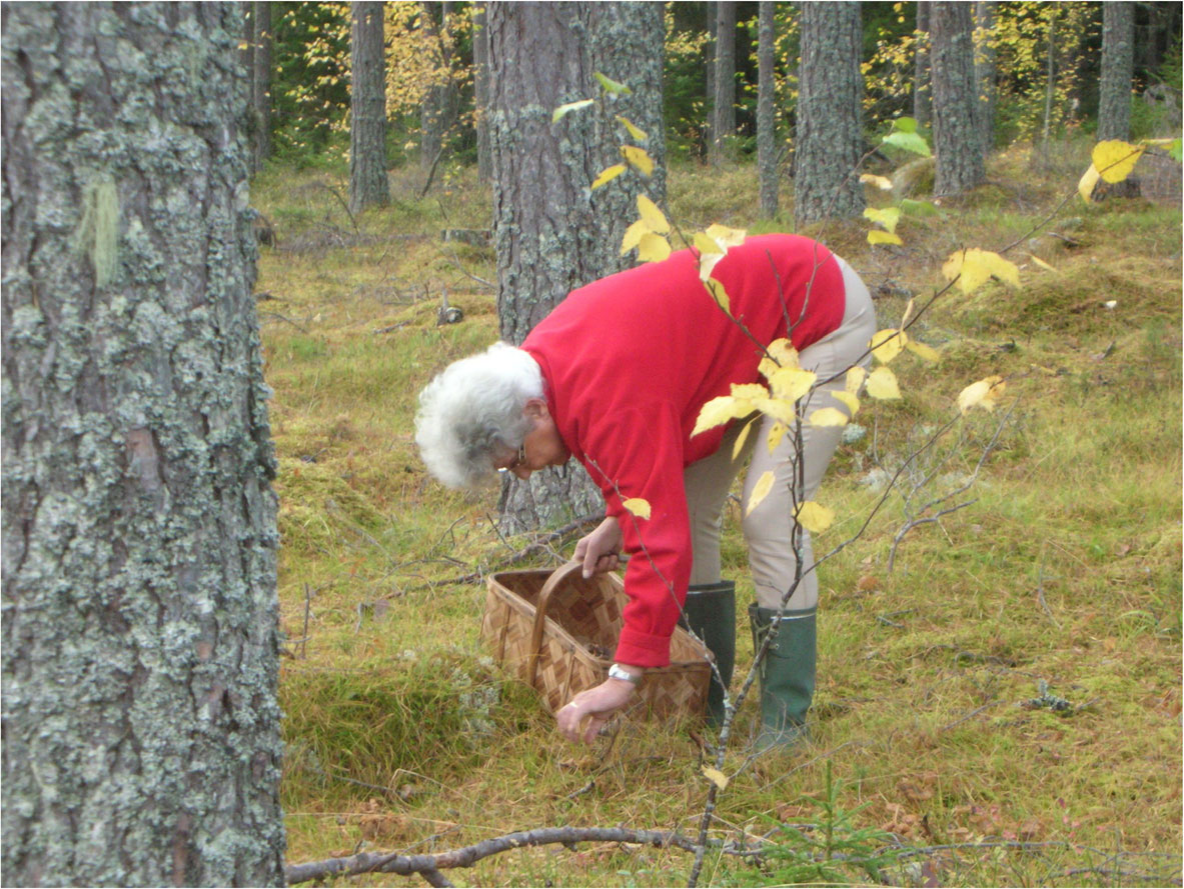
Foraging mushrooms is a popular recreational activity (photo: Maj Reinhammar)

In general, the locations where preferred mushrooms grow are found by accident. Still, experienced mushroom hunters are likely to know about the different fungi species’ preferred growing conditions, such as specific combinations of tree species, degree of moisture, and soil type, which is giving them guidance on where to forage. Many Swedish gatherers have certain mushroom spots (Swedish *svampställen*) which they return to each season (Fig. 9). As much as 64% of the study participants reported that they know of and use specific mushroom collecting spots. Areas suitable for fungi collection are preferably located on the countryside due to the much higher pressure on nature areas in the city surroundings.

One woman born in the 1990s reported that:

…I love the feeling of foraging my own food and knowing exactly where the raw materials originate from. I usually collect fungi from the countryside where I grew up since the rural areas are not as exploited by other foragers compared to the nature sites close to the cities…

Another woman, born in the 1960s, stated that:

I prefer to gather fungi in the woods that are surrounding my childhood home and there are special spots there…Since I hunt, I have a specific smartphone application in my cell phone that helps me finding my way home again. It gives me the opportunity of strolling without a concern and look for mushrooms everywhere if I want to…

These mushroom collection spots are almost always kept secret from others and often quite remote, making bikes and cars necessary in order to reach them. It is common to use small roads constructed for the forestry, and even though these paths often are shut off with locked bars, the foragers are still allowed to walk or bike in order to attain their collection areas. However, modern forestry is still a common obstacle for mushroom gathering. Many of the informants described that they had lost their favourite gathering spots due to the, often destructive, timber industry, creating clear-felled areas, alongside the expansion of human settlements.

One female informant, born in the 1940s, reported that:

…Some of my special spots have become devastated and ruined by the idiotic and incorrect “forest conservation”. The last time when I uploaded pictures of such an area on Facebook, a large group of people contacted me since they had similar experiences…

Another woman, born in the 1960s, stated that:

…All my old mushroom spots are clear cut now and therefore I’ve been looking for new collection locations that I will be able to return to in the future…

A male informant, born in the 1950s, reported that:

…My mother’s mushroom collecting spots do not exist anymore and without a car it is hard to find private favourite spots since the forests are very well-visited by other collectors. However, at my brother’s place in the countryside there are some good spots. I save the coordinates when I find fungi…¨

### Truffles

The interest for truffles, especially the earlier overlooked black truffle, *Tuber aestivum* Vitt., is increasing especially on Gotland. There is now a truffle academy, truffle conferences, education efforts, and yearly truffle festivals. Also, truffle dogs are now trained in Sweden. However, nothing of this was identified through the responses to our questionnaire [100].

### Mushroom clubs

**Fig. 10:**
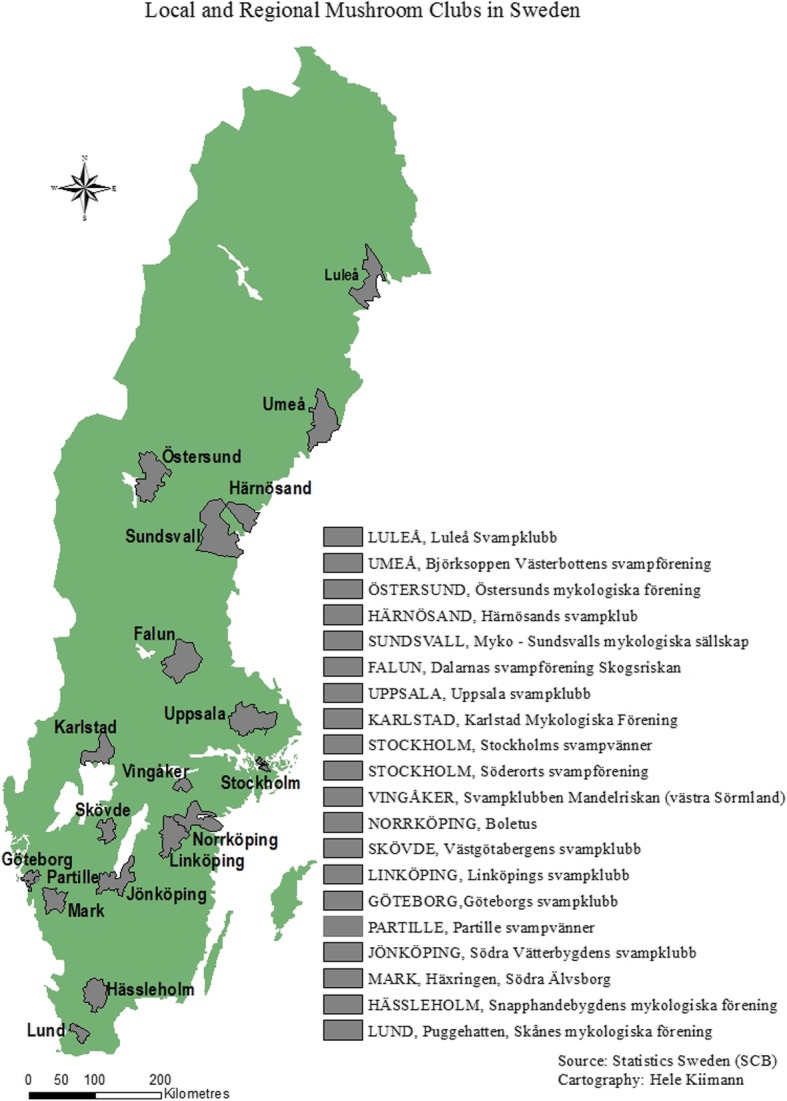
Local and regional mushroom clubs in Sweden

Today, there are many local and regional mushroom clubs in Sweden which organize meetings and excursion. There are at least 20 such clubs in the country (Fig. 10). The oldest, still functioning is Stockholm’s Svampvänner (Stockholm’s Fungi Friends’) founded since 1879 by a pupil to Elias Fries, mycologist Matts Adolf Lindblad. It was actually founded 5 years before Société mycologique de France and 16 years before Boston Mycological Club [101, 102]. However, most of the Swedish clubs are much younger. Uppsala Svampklubb (Uppsala Fungi Club) was founded in 1980 and has as its primary purpose the gathering and promotion of the mycological interests in the Uppsala area.

Some of these clubs have ordinary names, as the Uppsala Fungi Club, based on geographical background, while others have names after various fungi, such as *Häxringen* (‘Fairy Ring’), *Boletus*, *Mandelriskan* (=*Lactifluus volemus*), and *Björksoppen* (Birch Bolete) (Table 3).

**Table 3:** Swedish mushroom clubs (from south to north)

Puggehatten, Skånes mykologiska förening	
Snapphandebygdens mykologiska förening	
Häxringen, Södra Älvsborg	
Västgötabergens svampklubb	
Boletus, Norrköping	
Linköpings svampklubb	
Södra Vätterbygdens svampklubb	
Göteborgs svampklubb	
Partille svampvänner	
Svampklubben Mandelriskan (västra Sörmland)	
Karlstad Mykologiska Förening	
Stockholms svampvänner	
Söderorts svampförening	
Uppsala svampklubb	
Dalarnas svampförening Skogsriskan	
Myko - Sundsvalls mykologiska sällskap	
Östersunds mykologiska förening	
Härnösands svampklubb	
Björksoppen Västerbottens svampförening	
Luleå Svampklubb	

**Fig. 11:**
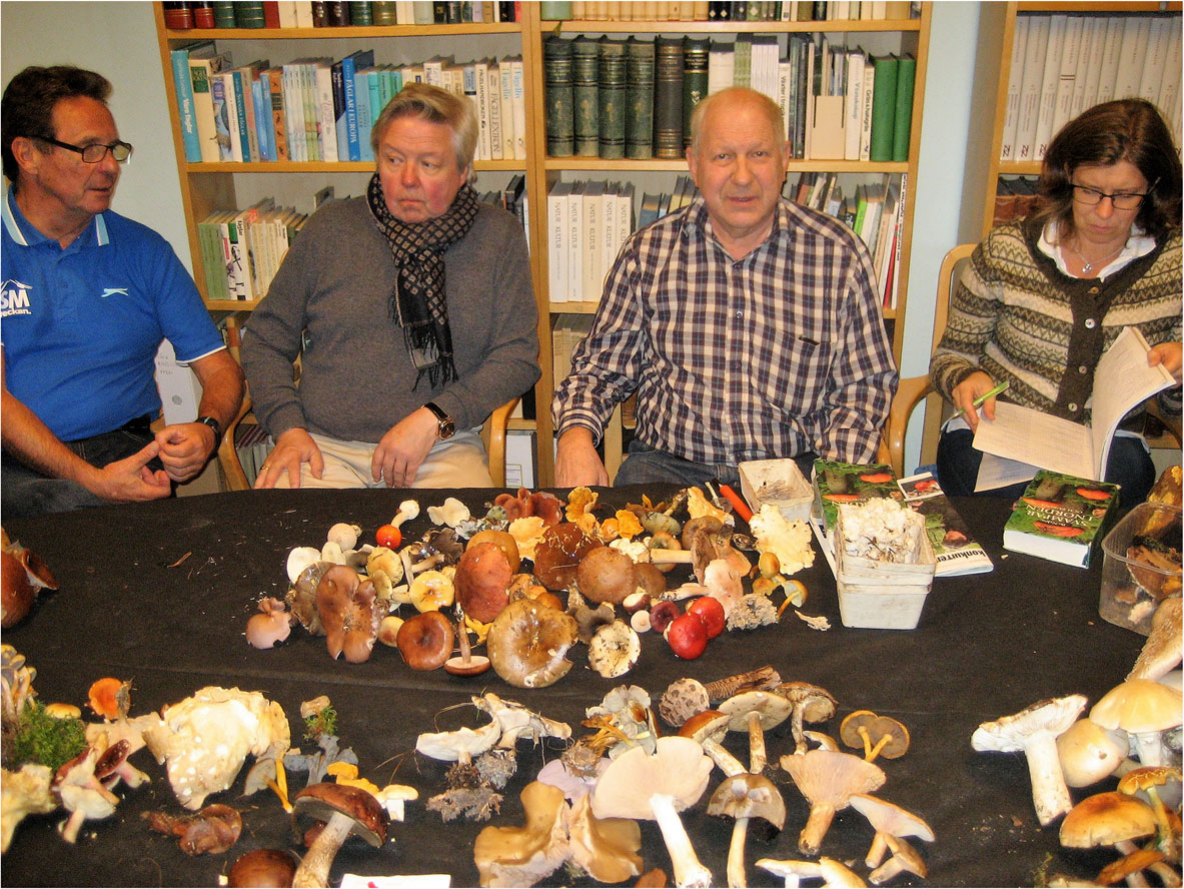
Members of the mushroom club ‘Boletus’ in Norrköping, identifying a fungi harvest at a weekly meeting (photo: Wiwi Emanuelsson, 10 October 2017)

A member of a mushroom club in Norrköping described the activities of her club (Fig. 11):

The club, which is called Boletus…lacks by-laws, member fees, managing board and everything else, which is normally part of a…society. It arises in the end of each summer season, when the first mushrooms show up and it lasts as long as there is still fungi to gather. Actually, it consists of a mailing list, which is being maintained by a driving spirit. In addition, we gain stability from the society for the conservation of Nature (Naturskyddsföreningen), which allows us to use their premises for free… Normally, we meet up at six o’clock in the evening on a suitable location in the natural surroundings of Norrköping. We walk in different directions through the woods and gather fungi during an hours’ time, before we get together and place our joint harvest on a large, green sheet. Thereafter, we collectively study the foraged mushroom material and create a list of the identified species. Later during the autumn, when it gets dark already by six o’clock, we meet indoors instead. Then everyone needs to forage their own fungi previous to the meeting and thereafter bring their harvest to the club…During winter time…we meet up a few times, mainly in order to deepen our theoretical knowledge…The number of club members is hard to estimate, but we are usually between eight and 12 people at each meeting.

### Fungi in social media

In later years, specific smartphone applications for identifying mushrooms have increased rapidly. Simultaneously, it has become common to upload pictures of one’s mushroom harvest on social media platforms in order to ask for advice regarding the gathered mushrooms’ identity. Still, there are individuals that would never ask for advice due to the pride in identifying ones harvest on their own.

For example, a woman born in the 1990s reported that:

I love to try out new types of fungi. We make cuts in them and identify them through books and smartphone applications. I never ask people on Facebook for advice, then there is no competition in it anymore.

As much as 74% of the Swedish internet users also use the social media platform Facebook on a regular basis. Another popular social media platform among the Swedes is Instagram, used by 53% of the population, according to the survey *Svenskarna och internet* (‘the Swedes and the Internet’) from 19 October 2017 [103].

The authors have observed how specific social media groups, created for people interested in fungi and the gathering of edible mushrooms, have increased rapidly for the past 5 years (Table 5). Furthermore, HL has witnessed a current mushroom gathering trend among young adults, especially the so-called hipster generation or Millennials [41], that involves documentation and dispersal of pictures taken during mushroom picking expeditions, through posts on Facebook and Instagram. These posts often include pictures of the gathering activity itself, the harvested fungi, the preparation of the harvest, and the resulting mushroom dominated meal.

A typical Facebook post involves a picture of the harvest, either in situ before gathering, inside a mushroom basket or back home at the kitchen table before preparation of the mushroom material. It is also common to add a picture of the actual mushroom forager, wearing open-air clothing, a mushroom basket, and sometimes a handbook for species determination. Pictures of a mushroom stew on a piece of bread are popular components of the perfect fungi Facebook post, often with a positive description, such as: ‘There’s a lot of mushrooms in the forest, now it’s time for chanterelle sandwich!’, ‘We brought five litres of fungi with us home from the woods, yummy!’ or ‘What a fantastic day in the forest! Now we are rich on berries and mushrooms!’ Often a hashtag is used within the describing text, such as #*skogensguld* (‘the gold of the forest’) or #*svamplycka* (‘mushroom happiness’) (Table 4).

**Table 4:** Fungi-related hashtags in social media

Hashtag	English translation	Frequency in social media
#*svampplockning*	Mushroom gathering	8060 public posts on Instagram 27 November 2017.
#*svamplycka*	Mushroom happiness/success	1527 public posts on Instagram 27 November 2017.
#*svamp*	Mushroom	94,093 public posts on Instagram 27 November 2017.

Numbers obtained during November 2017

HL has observed that younger social media users include pictures of themselves within these updates to a greater extent compared to older users. Among the younger age group, the so-called selfies (i.e. self-portrait photographs taken with a smartphone) together with the fellow mushroom gatherers are very popular. Among the social media updates on mushroom gathering (both on private user pages as well as within open groups for mushroom interested users), it appears as if the recreational significance of mushroom gathering expeditions is the most valued factor. People enjoy consuming edible mushrooms as a delicacy and an interesting add-up to the household pantry. Still, there is no personal disaster if the expedition results in a small or non-existent harvest. The typical Swedish mushroom gatherer collects fungi simply for the pleasure of spending time in the nature and due to the joy of consuming self-foraged, well tasting raw materials. Therefore, pictures from unsuccessful fungi gathering expeditions are also common, often with a positive comment on the weather or the company of other gatherers, which made it worth the effort despite the lack of mushrooms. In those cases, photographs of unidentified or inedible mushrooms are common, often with a comment, such as: ‘We found a lot of mushrooms, but no edible ones.’ The picture may also have been uploaded due to the interesting look of the photographed fungi, and in those cases, this is often commented in the written description. Above all, Swedes seem to value spending time in their natural environments and they simply cannot resist to slightly brag about their brisk walks in the woods in order to receive ‘likes’ and online comments from their social media followers. To be an active mushroom gatherer also means being healthy and admirable in many Swedish social contexts, and therefore, it comes as no surprise that the Swedes enjoy sharing their gathering experiences with their whole social media circle of acquaintances in order to get their longed for acknowledgements (Table 5).

**Table 5:** Number of members and updates in fungi-related social media groups

Name of group	English translation	Number of members	Average number of posts
*Svamp-klapp*	Mushroom clap	33,000	More than 10 new posts per day
*Färga garn med svamp och växter*	Colour wool with fungi and plants	3300	Two new posts per day
*Vilken svamp*	What mushroom	15,000	Four new posts per day
*Svampmat*	Mushroom food	2200	Two new posts per day
*Svampvänner*	Mushroom friends	3800	–
*Svampplockarna*	Mushroom gatherers	3500	New posts per week

Numbers obtained during November 2017

### Other uses of mushrooms

In our questionnaire, we also asked for other uses of mushrooms. We were interested in if their where any time-honoured uses of for instance brackets. In the 1980s, it became popular to dye wool with the help of specific mushrooms. A special society for promoting the interest in making dyes of fungi was founded in 1992, called Fungus Dye Society. Also, a couple of handbooks were published and courses were organized in many places. In the questionnaire, we specifically asked about the use of fungi as dye [14, 104]. However, the answers were few. A female informant born in the 1960s stated:

I have gathered fungi to dye wool. I have also gathered tinder bracket [= Fomes fomentarius] to make tinder.

A few informants (*n* = 3) also mentioned that they have gathered bracket fungi for decoration; one informant is still using smoke of burning tinder bracket as a repellent against mosquitoes. Another informant originating from rural Norrbotten remembered that her parents used the fruiting body of the birch conk, *Fomitopsis betulina* (Bull.) B.K.Cui, M.L.Han & Y.C.Dai, as needle pads. The latter uses have a long historical tradition in Sweden [14].

One male informant born in the 1950s reported:

my partner gathered fire spongs [= *Phellinus igniarius* (L.) Quél.] to keep the mosquitos away since we often camp. My son and I have produced touchwood out of tinder bracket [= Fomes fomentarius (L.) Fr.] and learned to make fire with it and fire steel.

Female informant born in the 1990s reported:

We have gathered bläcksvamp [ = Coprinus sp.] to dye with and smaller polypores in the northern part of Sweden for the mosquitos.

### Some closing notes

During the last century, the inhabitants of Sweden began changing from a mainly mycophobic culture to one willing to eat wild-harvested fungi species. The urban middle class, in particular, has accepted mushrooms increasingly in recent years. In spite of that, only an insignificant quantity of the edible macrofungi produced in Swedish forests is actually harvested [45]. Our research shows that few people who go mushrooming gather more than one or two species, mostly chanterelles, boletes, and a couple of other taxa. Avoiding poisonous mushrooms continues to be an important concern, so foragers gather only species they know with certainty. A dedicated few have become hobby specialists or connoisseurs and try to learn to identify as many edible species as possible.

Lay knowledge of native edible mushrooms is a direct result of education and information efforts. Members of mushroom clubs have better knowledge than others. Most people, however, gather mushrooms for recreational reasons, not for economic ones [105]. Picking your own mushrooms has also become part of the trendy finer home cuisine practiced by modern families. In contrast to Sweden (and the other Nordic countries), the people of Poland, who live south of the Baltic Sea, place a high cultural importance on fungi. Poland can be characterized as a mycophilous society, like Belarus, Russia, Ukraine [8, 42, 106], and most other eastern European (especially Slavic-speaking) countries [43]*.* Recently, scholars have identified a very high level of traditional knowledge about fungi in Poland’s Mazovia region [106]. Seventy-six species are being used there as food! Sweden has a long way to go to reach the level of its neighbours.

## Conclusion

The purpose of this paper is to study the acceptance of fungi as ingredients in Swedish cuisine and the growth of people’s interest in mushrooming. The Swedish population, although very urbanized, still has a close relationship with the forests. Some forest activities are increasing, such as hunting for elk, roe deer, and wild boar. The gathering of wild berries has decreased a bit in recent decades, while mushrooming has become increasingly popular. Extensive publicity and knowledge sharing especially in the 1970s and early 1980s, for harvesting fungi as food, has had a significant impact. Interest dipped after the Chernobyl disaster in April 1986, but in the last few years, mushrooming has started to regain its popularity. The overall impression in the twenty-first century is that mushroom gathering has become a common and appreciated recreational activity among Swedes. Many people have increased their knowledge through evening classes, guide books, membership in clubs, and other educational efforts and can now distinguish several edible species. Furthermore, modern aids (including social media) are being used to identify different types of fungi. Edible macrofungi can be a rich and varied ingredient in contemporary Swedish home-made food and restaurant cooking. One hundred years ago, most people rejected fungi as food. Today’s urbanized majority has learned to appreciate these forest delicacies in cooking. The people of Sweden have become mycophilic.

## Data Availability

The responses on the questionnaire are available at the Institute for Language and Folklore, Uppsala, Sweden: DFU 41174.

## Footnotes

1 Today, *Flugsvamp* is also a site name on Darknet or the dark web where illegal trade in hallucinogenic mushrooms and other drugs occurs. This domain of fungi use will not be dealt with in this article.

2 Scientific names are taken from Index Fungorum (www.indexfungorum.org, retrieved in 20 January 2018). However, *Cantharellus pallens* is considered as an own folk taxon among most contemporary mushroomers (Swedish *sommarkantarell*). The species has generally been defined as a species in its own right in Sweden until recently, but nowadays, it is considered to be a variety, *C. cibarius var. pallens* or, as in Index Fungorum, just a synonym for *C. cibarius*. For Swedish names, see https://svampguiden.com.
